# Promising Antimicrobial Properties of Bioactive Compounds from Different Honeybee Products

**DOI:** 10.3390/molecules26134007

**Published:** 2021-06-30

**Authors:** Magdalena Ratajczak, Dorota Kaminska, Eliza Matuszewska, Elżbieta Hołderna-Kedzia, Jarosław Rogacki, Jan Matysiak

**Affiliations:** 1 Chair and Department of Genetics and Pharmaceutical Microbiology, Poznan University of Medical Sciences, Swiecickiego 4, 60-781 Poznan, Poland; kamdor2@wp.pl; 2 Department of Inorganic and Analytical Chemistry, Poznan University of Medical Sciences, Grunwaldzka 6, 60-780 Poznan, Poland; eliza.matuszewska@ump.edu.pl (E.M.); jmatysiak@ump.edu.pl (J.M.); 3 Institute of Natural Fibres and Medicinal Plants, Wojska Polskiego 71b, 60-630 Poznan, Poland; elzbieta.kedzia@iwnirz.pl; 4 Department of Pediatric Surgery, Traumatology and Urology, Poznan University of Medical Sciences, Szpitalna 27/33, 60-572 Poznan, Poland; jrogacki@ump.edu.pl

**Keywords:** honeybee products, honey, propolis, bee venom, royal jelly, antimicrobial activity

## Abstract

Bee products have been known for centuries for their versatile healing properties. In recent decades they have become the subject of documented scientific research. This review aims to present and compare the impact of bee products and their components as antimicrobial agents. Honey, propolis, royal jelly and bee venom are bee products that have antibacterial properties. Sensitivity of bacteria to these products varies considerably between products and varieties of the same product depending on their origin. According to the type of bee product, different degrees of activity were observed against Gram-positive and Gram-negative bacteria, yeasts, molds and dermatophytes, as well as biofilm-forming microorganisms. *Pseudomonas aeruginosa* turned out to be the most resistant to bee products. An analysis of average minimum inhibitory concentration values for bee products showed that bee venom has the strongest bacterial effectiveness, while royal jelly showed the weakest antibacterial activity. The most challenging problems associated with using bee products for medical purposes are dosage and safety. The complexity and variability in composition of these products raise the need for their standardization before safe and predictable clinical uses can be achieved.

## 1. Introduction

Products of natural origin have been used in traditional medicine for centuries, and the beneficial properties of bee products have been known since ancient times [1]. Even though honeybee products have different chemical compositions, they exhibit similar properties, such as antibacterial, antifungal, antiviral, antiparasitic, anti-inflammatory, antiproliferative and antioxidant properties [2,3]. The composition of phenolics and flavonoids in honey and propolis is variable due to various geographical origins, types of honey floral plants and even climate [4,5]. Recent studies show that bee products from a variety of geographical origins and/or different floral sources exhibit different levels of antimicrobial activity [6,7]. Honey produced by *Apis mellifera* bees is one of the oldest bee products. The properties of honey in treating inflammatory diseases of the eyes, as well as for cleansing infected wounds and accelerating healing, were appreciated by the civilization of ancient Egypt. Hippocrates used honey in liver, stomach and intestinal diseases and for expectoration, strengthening and protection against infection [8]. In India, lotus honey was used to treat eye infections, among other things. In traditional medicine, it was applied to burns, skin diseases, ulcers, boils and insect bites [9]. Propolis (bee glue), in contrast to honey and royal jelly, has no nutritional value. Propolis is the resinous secretion of buds and young shoots of certain trees or the exudative substance produced by plants after they have been damaged, to which wax and small amounts of glandular secretion are added by bees. In addition, propolis may contain mechanical admixtures such as dust, pollen, fragments of dead animals and hive structures. These admixtures are removed during technological processing, and the ethanolic extract of propolis (EEP), obtained as a result of extraction, is a rich set of biologically active substances, including flavonoids, phenolic acids and their esters, lipid-wax substances, bioelements and others [1]. Therefore, propolis shows antibacterial, antioxidant, anti-inflammatory, antidiabetic, antiallergic, immunomodulatory and anticancer properties [10,11,12]. The anticancer potential ingredients have been identified as caffeic acid phenethyl ester, chrysin, artepillin C, galangin, etc. [13,14,15]. Caffeic acid phenethyl ester also has an antimicrobial effect against cariogenic bacteria [16]. Due to its biological properties, in practice people use it in the treatment of both skin diseases (especially microbial) and internal diseases [2]. Royal jelly (RJ), produced from the hypopharyngeal and mandibular salivary glands of young nurse bees, is an exclusive nourishment for the bee queen. This glandular secretion is white-yellowish, has a gelatinous–viscous sour taste and has been used since ancient times in caring for human health. It is still widely used, especially in Asia. RJ is susceptible to light and heat. It undergoes oxidation in direct contact with air. Aristotle attributed an increase in physical strength to the consumption of RJ and, above all, he suspected its role in improving intellectual capacity. Cleopatra used RJ as one of her personal beauty secrets. Chemically, it is a rich protein–carbohydrate–lipid product with a powerful cell- and tissue-stimulating effect. It exhibits immunostimulatory, anti-inflammatory, antitumor and antimicrobial effects [1,17]. The presence of antimicrobial properties of RJ with respect to Gram-positive and Gram-negative bacteria was scientifically demonstrated for the first time by McCleskey and Melampy in 1939 [18]. Bee venom (BV) has also been used for medicinal purposes since the time of ancient Egypt. It was used to treat skin maladies, back pain, multiple sclerosis and rheumatoid arthritis [19,20]. BV contains biologically diverse active compounds, including polypeptides (melittin, apamin, MCD peptide), enzymes (hyaluronidase, phospholipase A2) and biogenic amines (histamine, dopamine) [6,21]. Extensive exposure to antibiotics has led to the emergence and nationwide propagation of multidrug-resistant bacteria. This has necessitated the discovery and development of novel antimicrobial agents. Treating infections caused by bacteria that reside in the form of biofilms is equally problematic. They are characterized by greater resistance to the action of antibiotics [22]. The bacteriostatic and bactericidal activity of bee products against pathogenic microorganisms has been demonstrated in numerous studies [6,10,23,24]. This review aims to present and compare the impact of bee products and their components as antimicrobial agents.

## 2. Antimicrobial Properties of Bee Products

### 2.1. Honey

Honey has been shown to have an inhibitory effect on the growth of various species of Gram-positive and Gram-negative, aerobic and anaerobic bacteria. These properties are exploited in apitherapy, a growing branch of nonconventional medicine that uses bee products in the prevention and treatment of diseases. The antimicrobial activity of honey is attributed both to its physical properties (low acidity and high osmotic pressure) and to enzymatic factors (glucose oxidase, lysozyme) as well as chemical constituents (thermostable antibiotic substances: phenolic acids, flavonoids, benzoic acid, essential oils and their components, methylglyoxal). In an aqueous media, as a result of the glucose oxidase enzyme present in honey, the enzymatic decomposition of glucose to gluconic acid and hydrogen peroxide with antimicrobial properties takes place [9,25,26]. Recent studies have shown that increased temperature significantly reduces the bactericidal properties of tested honeys [23].

Research on manuka and tualang honeys has shown activity against bacteria such as *Escherichia coli*, *Enterobacter cloacae*, *Salmonella* Typhi, *Pseudomonas aeruginosa*, *Acinetobacter baumannii*, *Proteus mirabilis*, *Staphylococcus aureus*, coagulase-negative *Staphylococci* and *Streptococcus pyogenes* [27]. Interestingly, manuka honey is also effective against methicillin-resistant strains of *S. aureus* (MRSA) and vancomycin-resistant *Enterococcus* spp. (VRE) [9]. Tualang honey is more effective than manuka against some Gram-negative bacteria, probably due to its higher content of phenols, flavonoids and 5-hydroxymethyl furfural. It reduced the growth of *P. aeruginosa*, *A. baumannii* and *Klebsiella pneumoniae*, which were the cause of wound infections [9,27]. In addition, minimum inhibitory concentration (MIC) in the range of 8.75 to 25% for tualang honey [28] was found with respect to selected pathogenic Gram-positive and Gram-negative bacteria. The MIC of manuka honey assessed against clinical strains of *P. aeruginosa* was found to be between 10 and 20% [29]. However, MIC for ulmo (*Eucryphia cordifolia*) honey had a lower value (3.1–6.3%) than for manuka honey (12.5%) for MRSA isolates.

The antibacterial potential of honey, in particular manuka and tualang, is known worldwide, and honey can be used as an alternative therapeutic agent. Low concentrations of tualang honey inhibit the growth of *S.* Typhi, *Shigella flexneri* and *E. coli* bacteria, responsible for foodborne infections. As such, taken orally, in an undiluted form, it can shorten the duration of diarrhea. Honey has also been shown to inhibit the growth of strains such as MRSA and multidrug-resistant *P. aeruginosa* and *Acinetobacter* spp., as well as those from the *Enterobacteriaceae* family that cause burn wound infections and hemolytic uremic syndrome [30,31]. In addition, manuka and capilano honeys have been shown to inhibit the growth of *Helicobacter pylori* [9]. Good results for honeys (Sudanese, Nigerian, New Zealand, Indian, Slovakian and Polish) were obtained against aerobic Gram-positive (*S. aureus*, *Enterococcus* spp.), Gram-negative (*P. aeruginosa*, *Stenotrophomonas maltophilia*) and anaerobic (*Clostridium adenomaticus*) bacteria, among others. The strongest effects (2.0–14.1%) were demonstrated by New Zealand honeys (manuka and meadow honey) and Slovakian conifer honeydew honey (9.5%) [23,32,33].

The impact of honey on different fungal species was also evaluated. Khosravi et al. [34] demonstrated that *Candida glabrata*, *Candida tropicalis* and *Candida dubliniensis* strains proved to be the most sensitive, with average MIC values of 38.3% *v/v*, 39.3% *v/v* and 37.8% *v/v*, respectively, while *Candida kefyr* and *Candida albicans* strains showed greater resistance to the tested honeys. Higher concentrations of honey reduced the growth of *Candida*. All honeys entirely inhibited the growth of the tested yeast spores at minimum fungicidal concentrations values between 29 and 56%.

The antifungal activity of honeys against dermatophytes was tested by an agar diffusion test. The results indicated that agastache honey at a concentration of 40% was most effective against *Trichophyton mentagrophytes* and *Trichophyton rubrum.* Its activity against *T. mentagrophytes* was between 20 and 10 mm, and its activity against *T. rubrum* was between 19.5 and 12 mm. A weaker effect against dermatophytes was found for tea tree honey. Manuka honey showed weak activity against *T. mentagrophytes* and no activity against *T. rubrum*. Other tested honeys, namely jelly bush, super manuka and jarrah, did not show antifungal activity against any of the tested dermatophyte isolates [35].

It has been suggested that honey may act synergistically with antibiotics. The effect of oxacillin and manuka honey on MRSA strains has been described [36]. Müller et al. proved the effect of manuka honey in combination with rifampicin against clinical MRSA isolates. Furthermore, the synergistic effect of oxacillin, clindamycin and rifampicin against *S. aureus* strains was demonstrated by combining these antibiotics with honey [36].

These experiments provide scientific evidence of antimicrobial activity of different varieties of honey from remote areas of the world [5]. The activity of different honeys against microorganisms is shown in Table 1. Due to the many factors that influence the antibacterial properties of honeys, the difference in their antimicrobial effects can be over 100-fold [9]. In general, the nonstandardized, unpredictable antimicrobial activity of honeys hinders their introduction as an antimicrobial agent. Currently, the best known with specific antibacterial properties in vitro are manuka and tualang honeys [9,27,28]. The medical-grade honey has the potential to be topical antibacterial prophylaxis because of its broad-spectrum bactericidal activity.

### 2.2. Propolis

Numerous studies have shown that propolis can inhibit the growth of bacteria and has antifungal properties [40,41,42,43,44,45,46,47]. Raw propolis cannot be used directly in a treatment. First, it must be extracted in order to dissolve and release the most active ingredients. The following solvents are used as extractants: ethanol, methanol, water, hexane, acetone, dichloromethane and chloroform. Extracts contain a propolis concentration of approximately 70% [6]. In terms of antibacterial activity, the content of substances such as flavonoids and phenolic compounds is important. However, depending on the solvent used, different biological activity is found. Karpiński et al. [6] compared the activity of different propolis extracts against Gram-positive and Gram-negative bacteria. The most commonly used is an ethanolic extract of propolis (EEP). The MIC values for EEPs from different geographical origins against Gram-positive and Gram-negative bacteria were presented [6]. Analysis of the propolis mechanisms allows inferences regarding its effect on the permeability of the cellular membrane of microorganism, disruption of membrane potential and adenosine triphosphate (ATP) production, as well as on decreasing bacterial mobility [2,6].

Al-Ani et al. [48] demonstrated that all tested extracts from European countries were effective against Gram-positive bacteria, with an MIC range of 0.08 to 5.0 mg/mL. The strongest antimicrobial effect was observed against *Streptococcus* and *Bacillus subtilis.* In contrast, propolis had the weakest effect on *Enterococcus casseliflavus* and *Staphylococcus saprophyticus.* Furthermore, it showed moderate efficacy against MRSA strains (MIC between 0.3 and 1.2 mg/mL) and poor efficacy against vancomycin-resistant enterococci (MIC between 2.5 and 5.0 mg/mL). Wojtyczka et al. [49] also demonstrated that an ethanolic solution of European propolis exhibits antimicrobial activity against *Staphylococcus* spp. At a concentration of 0.76 mg/mL during a 12 h incubation, this solution caused growth inhibition of *S. aureus* strains. Other European researchers have also obtained similar results for *Staphylococcus and Enterococcus* Gram-positive bacteria [46]. However, Cardoso et al. [50] obtained different values by showing that the minimum bactericidal concentration of the EEP for *S. aureus* strains is about 13.3 mg/mL. In contrast, ethanol extract of Brazilian red propolis Alagoas inhibits the growth of *S. aureus* only at concentrations between 25 and 100 mg/mL. Moreover, it showed no activity against Gram-negative bacteria [44].

Most EEPs exhibited moderate bacteriostatic efficacy against Gram-negative microorganisms with MICs ranging from 0.24 to >5.0 mg/mL, while high resistance to propolis was exhibited by *E. cloacae, Salmonella choleraesuis* (MIC > 5 mg/mL) and *K. pneumoniae* strains [48]. Data are shown in Table 2.

Similarly, Schmidt et al. [53] demonstrated that the activity of propolis extract against Gram-negative bacteria was ineffective (MIC > 4000 µg/mL).

Studies indicate that ethanolic propolis preparations of appropriate concentration, composition and duration of action may be an alternative way to support therapy of diseases caused in particular by Gram-positive bacteria and may complement antibiotic therapy. Researchers confirm the synergistic effect of EEP with antibiotics. An increase in antimicrobial activity of some combinations of 10% EEP with topical antibiotics was demonstrated against *S. aureus* strains isolated from abscesses and infected wounds. The strongest effects were observed with gentamicin, oxytetracycline and bacitracin [55]. The synergistic effect of EEP from Ireland with vancomycin and oxacillin against MRSA strains has been demonstrated. Cooperation between propolis and vancomycin against *S. pyogenes* has also been reported. Furthermore, a combination of propolis and levofloxacin showed a more potent effect against *Streptococcus pneumoniae* and *Haemophilus influenzae,* microorganisms causing respiratory infections [48]. The synergistic effect of propolis and neomycin was confirmed by Orsi et al. [56]. Wojtyczka et al. [49] described an interaction of propolis with chloramphenicol, gentamicin, netylmycin, tetracycline and linezolid against Gram-positive bacteria. Other researchers have observed a synergistic effect of propolis with vancomycin and oxacillin against *S. pyogenes*.

The phenomenon of increasing resistance bacteria to antibiotics also applies to fungi [57]. Therefore we are looking for other alternative treatments for fungal infections. Many authors describe the antifungal activity of propolis [46,58]. Al-Ani et al. [48] showed that all samples of European propolis have antifungal properties against strains of the genus *Candida*, with MIC values between 0.1 and 5.0 mg/mL. Propolis from Ireland had the most effective fungicidal effect, with minimum fungicidal concentration (MFC) between 0.1 and 0.6 mg/mL. Propolis from Germany showed the weakest effect on yeast cells (MFC > 5 mg/mL). This study showed that *C. glabrata*, *C. parapsilosis*, and *C. tropicalis* were the most sensitive *Candida* species [48].

An in vitro study on yeast strains isolated from blood showed that Turkish propolis exhibits antifungal activity comparable with fluconazole and itraconazole [59]. The MFC values of propolis at which the growth of 50% and 90% of *C. albicans* isolates were inhibited were 0.375 and 0.75 µg/mL, respectively. Different results were obtained by Wojtyczka et al. [49]. They determined that an ethanolic extract of propolis in high concentrations (6250 µg/mL) completely inhibited the growth of *C. albicans* strains only after 24 h of incubation. Other researchers from Turkey have shown that native propolis is highly effective against *C. albicans* yeasts isolated from blood. *C. glabrata* proved to be the most resistant strain. The same authors tested the effect of propolis on 13 strains of *Trichosporon* dermatophytes, and MFC for these strains was between 0.0125 and 0.1 µg/mL [60]. Furthermore, Pepeljnjak and Kosalec [61] found that propolis at a concentration of 15–30 mg/mL inhibited the growth of *C. albicans, Aspergillus flavus, Aspergillus ochraceus, Penicillium viridicatum* and *Penicillium notatum.* Melliou et al. [62] tested the activity of propolis against Gram-negative and Gram-positive bacteria and *C. albicans, C. tropicalis and C. glabrata* yeasts. They observed the highest activity of propolis against *Candida* yeast-like fungi.

To sum up, we observed a wide variation in the antimicrobial activity of propolis. It is more effective against Gram-positive *Staphylococcus* and Gram-negative *Neisseria* than against Gram-positive *Enterococcus* and Gram-negative *E. coli* and *P. aeruginosa.* The high flavonoid content of bee putty inhibits the growth of *Candida* and dermatophytes. The flavanol galangin inhibits the growth of the following molds: *Aspergillus tamarii, A. flavus*, *Cladosporium sphaerospermum* and *Penicillium digitatum* [43,46,47,48,53,61]. The demonstrated differences in the activity of propolis may be due, among other things, to differences in chemical composition, origin, solvent used, extraction method and time and percentage of biologically active compounds [6]. The latest comparative studies show that propolis extracts at concentrations of 100 mg/mL, with propolis prepared using both ethyl alcohol and propylene glycol as solvents, have high antibacterial activity against *S. aureus and C. albicans* strains (MIC 2.0 and 2.5 mg/mL respectively) They showed weaker activity against *E. coli* (MIC = 100 mg/mL) [63]. Interesting results were obtained comparing the activity of propolis and its isolated ingredients. The lowest concentration of propolis inhibiting *S. aureus* growth was 0.80 mg/mL, the MIC of totarol was 0.07 mg/mL and the MIC of pinocembrin was 0.25 mg/mL. These results may explain the differences in activity of EEPs from different geographical regions; e.g., Brazilian propolis is rich in galangin and pinocembrin and showed strong antibacterial and antifungal activity [62], and Chilean propolis showed a level of activity against *Streptococcus mutans* that was higher than that of a mixture of polyphenols or even chlorhexidine [64].

Furthermore, we observed that propolis samples from different regions of the world and differing significantly in chemical composition are similar in their antibacterial, antifungal and antiviral activity. Although the extraction of propolis with ethanol is a simple and effective method, it may pose some limitations related to its practical use, e.g., in some branches of the cosmetic and pharmaceutical industries. In medicine, ethanol extracts are contraindicated mainly in ophthalmology, otolaryngology, pediatrics and diseases of the oral cavity [45]. Therefore, recent research has focused on testing the microbial activity of propolis prepared in other alcohol-free solvents, such as propylene glycol, glycerol, dichloromethane, hexane and supercritical fluid [28,42,47].

### 2.3. Bee Venom

Various antimicrobial peptides (AMPs) derived from the venom of different bee species have been reported: melittin, mastoparan, melectin, apamin, secapin and others [20,48]. Unfortunately, most of them, apart from their antibacterial activity, have a cytotoxic effect on mammalian cells. AMPs display antimicrobial activity usually through disrupting the bacterial membrane. These peptides have variable length and a positive charge which allows for an electrostatic interaction with negatively charged bacterial membranes [65]. AMPs can replace divalent cations such as Mg^2+^ and Ca^2+^ bound to LPS, causing membrane disruption and leading to the death of the bacteria [66]. Some AMPs can penetrate the bacterial membrane and kill bacteria without inducing bacterial membrane permeabilization. They can bind DNA, RNA and proteins and inhibit the synthesis of different functional and structural proteins [65].

The antibacterial activity of bee venom has been demonstrated against various strains of Gram-positive bacteria: *S. aureus* [67,68,69], *Staphylococcus hyicus* and *Staphylococcus chromogenes* [69], *Streptococcus salivarius, Streptococcus sanguinosus*, *Streptococcus sobrinus*, *Streptococcus mitis*, *S. mutans*, *E. faecalis* [70] and *B. subtilis* [48]. Its activity against Gram-negative bacteria has also been proven: *K. pneumoniae* [48], *Salmonella* Typhimurium and *E. coli* [71]. Gram-positive bacteria have been shown to be more susceptible to bee venom than Gram-negative bacteria [48,70] (Table 3).

Melittin is a major component of European honeybee *Apis mellifera* venom. In 1967, Fenell et al. demonstrated its activity against a wide variety of Gram-negative (*P. aeruginosa, S. maltophilia, Acinetobacter lwoffii, Enterobacter aerogenes, E. cloacae*, *Salmonella enterica* serotype Newport) and Gram-positive bacteria (*E. faecalis, Corynebacterium* spp., *S. aureus*) [88]. In the following years, there were more and more reports on the antibacterial activity of melittin. In vitro studies have demonstrated its antibacterial activity against a wide range of microorganisms [19,73,89,90]. In vivo studies also provide evidence for the antibacterial activity of melittin against *Chlamydia trachomatis* [91], *Cutibacterium acnes* [92], MRSA [49] and extensively drug-resistant (XDR) *A. baumannii* [93]. Antibacterial properties of BV and melittin seem promising, especially for multidrug-resistant (MDR) bacteria [48]. Various researchers have demonstrated the activity of bee venom against MRSA strains [48,89].

Encored by the results of studies on the antibacterial activity of bee venom and melittin, researchers started to combine them with one or more drugs in order to treat resistant bacterial infections. The effect of synergistic action against different strains of resistant bacteria has been achieved by many scientists. Antimicrobial activity of melittin in combination with doripenem and ceftazidime has been observed against *P. aeruginosa* MDR strains [94]. Promising results were also obtained by Al-Ani et al. [48] by comparing the effects of bee venom and melittin in various combinations with antibiotics and plant secondary metabolites against MDR bacteria. Han et al. [89] demonstrated BV antimicrobial and synergistic effects in combination with ampicillin, penicillin, gentamicin or vancomycin. The MIC values for BV were 0.085 and 0.11 µg/mL, respectively, for the two MRSA strains tested. The best bactericidal effect was obtained using BV in combination with gentamicin and vancomycin [89]. Although the results are promising, the activity of BV against more MRSA strains, as well as its synergistic effects with other antimicrobial substances, should be studied.

Melectin is a new antimicrobial peptide isolated by Cerovský et al. [95] from the venom of the cleptoparasitic bee *Melecta albifrons*. Melectin binds to LPS or LTA through electrostatic interactions and leads to the rapid death of bacteria through bacterial membrane permeabilization. The authors demonstrated antimicrobial activity of synthetic melectin against both Gram-positive and Gram-negative bacteria and its low hemolytic activity. Broad-spectrum antimicrobial activity of melectin, low cytotoxicity and no hemolytic activity were also confirmed by other authors [65,74]. The MIC measurements against *S. aureus*, *P. aeruginosa*, *S*. Typhimurium, *K. pneumoniae*, *E. coli* and drug-resistant bacteria were determined [65]. MIC was 2 μM against *S. aureus* and *P. aeruginosa* and 4 μM against *K. pneumonia* and *E. coli*. The antibacterial activity of melectin against the tested resistant strains of *S. aureus*, *P. aeruginosa* and *E. coli* was also confirmed (MIC = 2–8 μM). The authors demonstrated a similar antibacterial effect of melectin and melittin. In contrast, melectin has lower cytotoxicity and no hemolytic activity compared to melittin [65]. This peptide has a high potential for further research and application as a new drug.

Both bee venom and melittin derived from bee venom show varying antifungal activity against different species of pathogenic fungi (*T. mentagrophytes*, *T. rubrum* [96], *C. albicans* [48,87], *C. krusei*, *C. parapsilosis*, and *C. tropicalis* [48]). Lee demonstrated antifungal activity of BV against 10 clinical strains of *C. albicans*, with MFC values between 62.5 and 125 µg/mL [66]. Another study showed antifungal activity of BV and melittin against different yeast species (*C. albicans, C. parapsilosis, C. tropicalis* and *C. krusei*) with MFC values between 30 and 300 μg/mL [48].

Bee venom shows antibacterial and antifungal activity and synergistic effects with other antimicrobials, increasing their effectiveness against MDR bacteria. However, the strong toxic effect of the main active ingredient melittin against mammalian cells [65,97] hinders the possibility of using this substance for treatment. Current efforts by researchers focus on trying to reduce the toxicity of melittin without affecting its bactericidal activity. One of the proposed solutions to increase safety is to use it in the form of nanoparticles [98]. Another strategy involves coupling melittin with aptamers [99]. Combining natural melittin with antibiotics is another way to minimize its dosage, thereby reducing both concerns about its cytotoxicity and the likelihood of antibiotic-resistant strains developing.

### 2.4. Royal Jelly

RJ is composed of water; proteins; lipids; carbohydrates; and other substances, including organic acids, nucleic acids, nucleotides, hormones, pteridines and small amounts of vitamins and mineral salts [100,101]. The antimicrobial activity of RJ is mainly attributed to its constituent peptides such as major royal jelly proteins, royalisin, jelleines, apismin, royalectin and apolipophorin III-like proteins and fatty acids (10-HDA) [77,102,103]. 10-Hydroxy-2-decenoic acid (10-HDA), royalisin and jelleines have the greatest antimicrobial potential [102,103,104].

Garcia et al. [77] studied the antimicrobial effects of RJ, as well as their defatted and lipid extracts, from four different geographical areas in Argentina. They evaluated antimicrobial activity against bacteria that may be responsible for skin wound infections in humans and animals: *S. aureus* (including MRSA), *S. epidermidis*, *Micrococcus luteus*, *Streptococcus uberis*, *Streptococcus agalactiae*, *Streptococcus dysagalactiae*, *E. faecalis*, *E. faecium*, *P. aeruginosa*, *E. coli* and *K. pneumoniae*. Overall, researchers observed higher RJ and 10-HDA activity against Gram-positive bacteria than Gram-negative bacteria. Among Gram-positive microorganisms, bacteria belonging to the *Staphylococcus* genus were more sensitive to RJ than those of *Enterococcus* and *Streptococcus.* Examining the composition of the analyzed samples, the authors found that the observed antimicrobial activity is mainly due to the presence of 10-HDA [56]. Yang et al. [102] also showed high antimicrobial activity of 10-HDA against Gram-positive (*S. aureus, Streptococcus agalactolyticus, Staphylococcus intermedius, Staphylococcus xylosus*) and Gram-negative (*S. choleraesuis, Vibrio parahaemolyticus* and *E. coli* (hemolytic)) bacteria.

Bilikova et al. [81] compared the antibacterial activity of royalisin and its recombinant form royalisin-D truncated by 11 amino acid residues at the C-terminus. Both peptides showed similar activity against the nine microorganisms tested (*S. aureus*, *S. agalactolyticus*, *S. intermedius*, *S. xylosus*, *Paenibacillus larvae*, *P. aeruginosa*, *S. choleraesuis*, *V. parahaemolyticus*). All bacteria except *E. coli* showed sensitivity [81].

Jelleines (jelleines I, II, III and IV) are the other antimicrobial peptides identified in royal jelly. Fontana et al. [105] examined their antimicrobial activity against eleven bacterial species and the *C. albicans* yeast. Jelleines I and II manifested antibacterial activity against most Gram-positive and Gram-negative bacteria species and yeast. Jelleine-III had a reduced spectrum of activity, while jelleine-IV showed no antimicrobial activity [105]. Other scientists have also demonstrated the antimicrobial activity of jelleines [106,107,108,109]. Recently, Jia et al. [109] investigated the mechanism of action of jelleine-I against Gram-negative and Gram-positive bacteria and its antimicrobial activity in vivo. The researchers have shown that this peptide acts as an antimicrobial mainly by disrupting the integrity of the cell membrane, but also acts inside cells (genomic DNA). In an in vivo experiment on a mouse model, they showed that jelleine-I exerted a good therapeutic effect on mice with peritonitis caused by *E. coli* [109]. Promising results confirming in vivo antimicrobial activity of RJ were also obtained by Gunaldi et al. [110]. The authors conducted an evaluation of RJ performance in an implant-related infection model in rats. RJ did not prevent MRSA infection but markedly reduced its severity in a group of spinal implant rats inoculated with bacteria and treated with RJ, compared with untreated rats [110].

## 3. Comparison of Antimicrobial Properties of Bee Products

**Figure 1:**
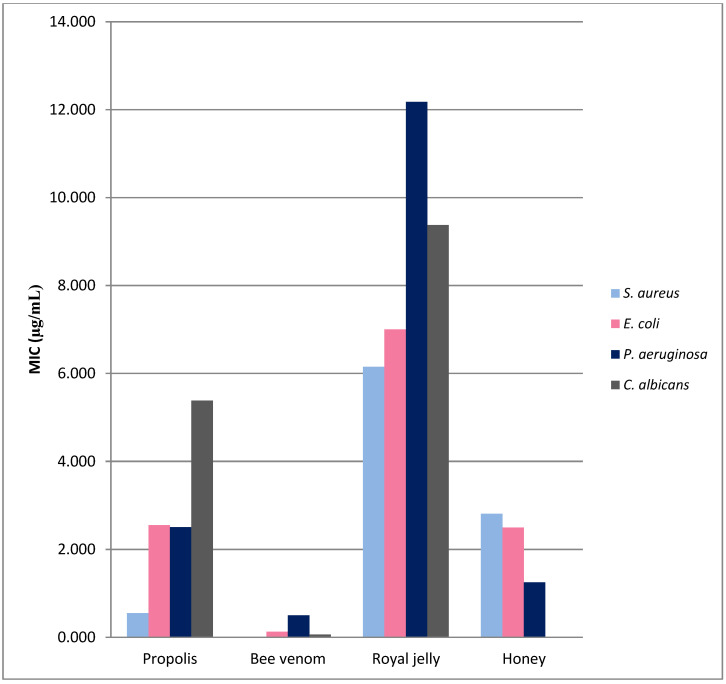
Antimicrobial activity of bee products. The graph shows the arithmetic mean MIC values for the most commonly tested microorganisms. Only experiments in which the MIC value was determined were included in the analysis. There are significant differences in the effects of individual bee products on different bacteria (propolis 10-fold, BV 35-fold and RJ 2-fold differences). BV showed the best antibacterial activity against all analyzed microorganisms (mean MIC values in the range from 13.98 µg/mL (*S. aureus*) to 500 µg/mL (*P. aeruginosa*)).

Analysis of literature in this review includes bee product MIC values for different microorganisms (Table 1, Table 2 and Table 3). Figure 1 depicts the collected results (arithmetic mean values). An analysis of the average MIC values for bee products confirmed their higher efficacy against Gram-positive than against Gram-negative bacteria. The highest MIC values, and therefore the weakest antimicrobial activity, were recorded for royal jelly. *P. aeruginosa* turned out to be most resistant to bee products. The highest antibacterial activity was observed for bee venom and melittin. The best activity of BV was observed for *S. aureus* (average MIC 13.98 µg/mL), while BV was the least active on *P. aeruginosa* (MIC average of 500 µg/mL). The use of melittin and other AMPs from bee venom in combination with drugs (amikacin, ceftazidime, imipenem and ciprofloxacin) also showed bactericidal and synergistic effects. In addition, the combination of a natural product and a commercial drug has great potential for use as a rapid and effective method of treatment for serious infections. Furthermore, together they may have a more rapid bactericidal effect and perhaps lessen the effects of bacterial resistance. AMPs from bee products show very promising antimicrobial properties; however, it should be remembered that they often also have a strong cytotoxic effect.

Different authors use various methods to test antimicrobial activity, which makes it difficult to compare results. Therefore, only results for which MIC was determined by serial dilution method are included in the analysis presented here. Different MIC values for bee products observed in experiments with the same genus of bacteria can be explained by (I) the difference in the composition and concentration of active substances, (II) the sensitivity of the strain tested, (III) different durations of action of bee products on the tested microorganisms and (IV) the method used to evaluate bioactivity.

The composition of bioactive compounds of bee products is variable due to various geographical origins. Selected bioactive compounds responsible for the antibacterial activity are presented in Table 4.

## 4. Prospective Use of Bee Products in the Treatment of Biofilm-Related Infections

Infections associated with biofilm formation are especially difficult to treat. Bacterial biofilms are more resistant to antibiotics than planktonic forms. Therefore, substances are needed to combat biofilms, for which therapy with available drugs is often ineffective.

Manuka honey has been proven to have anti-biofilm effects [111]. The authors found that honey reduces biofilm mass by destroying bacterial cells trapped in its matrix. They studied the effects of New Zealand manuka honeys on *P. aeruginosa* strains with different biofilm-forming abilities. They showed that honey at concentrations of 64% and 80% inhibited the adhesive ability of strains and reduced the biofilm formed [111]. Other researchers used a combination of manuka honey and antibiotics to treat chronic infections with biofilm formation and found that the combination with rifampicin was most effective against staphylococcal biofilms. Interestingly, some combinations of antibiotics and honey showed antagonistic effects (gentamicin and oxacillin) and others such as fusidic acid and clindamycin showed synergistic effects against *S. aureus* biofilm [36].

For activity against biofilms, analogs of natural antifungal peptides (AFPs) originally isolated from bee venom were also tested: LL-III (LL-III/43) and HAL-2 (peptide VIII). According to studies, both peptides can be used to inhibit biofilm produced by *Candida* spp. A decrease in the area colonized by biofilms was observed, as was an inhibition of the production of filaments. Moreover, compared to currently used antifungal drugs, AFPs show low hemolytic and cytotoxic activity. The use of these newly synthesized compounds may lead to the solution of an important medical problem associated with *Candida* biofilm infections [37].

Several studies have examined in vitro the effect of melittin on the viability of bacteria in biofilm form [112,113,114]. It has been shown to be active against clinical isolates of biofilm-producing *P. aeruginosa* (MBIC between 4 and 16 μM) [114]. In addition, Bardbari et al. determined the ability of melittin, or its combinations with colistin and imipenem, to inhibit MDR strains of *A. baumannii* producing a strong biofilm [113].

Valuable information on bacterial adhesion, which plays an important role in the initial phase of biofilm formation and development of infections, was provided by da Cunha et al. [115] and Susilowati et al. [116]. Da Cunha investigated the effect of geopropolis, collected from *Melipona scutellaris*, a species of stingless bees found in tropical countries, on *S. mutans* biofilm. They found that propolis inhibited bacterial cell adhesion. Furthermore, other geopropolis samples showed similar mechanisms of action against *S. mutans*, reducing cell viability in the biofilm [115]. Susilowati et al. investigated the effect of RJ on *P. aeruginosa* adhesion to an abiotic surface and human pharyngeal and lung epithelial cell lines (Detroit 562 and NCI-H292), as well as the anti-inflammatory effect of RJ on the above *P. aeruginosa-*stimulated epithelial cells [116]. In the experiment, royal jelly did not show antimicrobial activity at a concentration of 50% *w/v*, while antiadhesive activity was observed on the abiotic surface and epithelial cells at a concentration of 25%. The mechanisms of RJ acting on *P. aeruginosa* adhesion inhibition are unknown [116].

Bee products show antibiofilm potential and could be used to develop new alternative treatments for various infections associated with biofilm formation. Wound dressings soaked in manuka honey can be used in supportive therapy for infected chronic wounds, including those containing *P. aeruginosa* biofilms [111]. It is worth noting that industrially produced dressings with medicinal manuka honey (Medihoney, Beaudesert, Australia) have been used successfully in clinical settings for the treatment of wounds and ulcers since the beginning of this century. Propolis, on the other hand, in appropriate concentrations can be used as an anticaries agent. Furthermore, analogs of natural antifungal peptides isolated from BV may find application in inhibiting fungal colonization, in particular in preventing vulvar and vaginal infections associated with *Candida* spp. biofilm [115].

## 5. Advances in the Characterization of Bee Products’ Antibacterial Properties

Despite the undoubted benefits of bee products in medicine, it should be noted that these products are complex biological matrices whose composition has not been fully characterized yet. Moreover, until now, the complete mechanism of their antimicrobial action has not been investigated. Therefore, modern analytical methods must be applied to broaden the knowledge of bee products’ activities and composition, which will enhance the safety of their usage for medical purposes [116,117]. According to available literature, methods used for searching for active compounds in bee products are based mainly on spectroscopy, spectrophotometry and mass spectrometry. Separation techniques such as gas chromatography, high-performance liquid chromatography and capillary electrophoresis are also applied in experiments [118]. 

The Fourier transform mid-infrared spectroscopy equipped with attenuated total reflectance (FT-IR–ATR) method was used by da Silva et al. [119] to quantify total phenolic contents in propolis extracts. Since a phenolic fraction plays an important role in the antibacterial properties of bee products, these compounds have been extensively studied both quantitively and qualitatively using spectrophotometry [120,121,122], high-performance liquid chromatography (HPLC) [123,124,125], gas chromatography [126] and mass spectrometry [127,128,129,130,131], as well as in combinations. Moreover, advanced techniques are used for the determination of other antibacterial constituents, such as methylglyoxal [132,133] or 10-hydroxy-2-decenoic acid (10-HDA) [130]. The most commonly used methods for the analysis of selected antibacterial are shown in Table 5.

Antibacterial properties of bee products may be estimated not only by direct analysis of active compounds contained in them. An interesting approach is a characterization of the processes occurring in bacteria exposed to beehive products. Packer et al. [131], using two-dimensional gel electrophoresis (2-DE) and LC-MS, examined the proteome of *S. aureus* grown with the addition of manuka or jelly bush honey to the microbial culture. The results indicated that honey has a significant effect on the *S. aureus* proteome. Proteins involved in amino acid or protein synthesis and energy metabolism were downregulated. On the other hand, upregulation of stress proteins was noticed. Moreover, honey’s interference with the ribosome or its translational capacity has been observed [134,135]. These results prove the antibacterial activity of honey and its potential value as an antibacterial agent.

## 6. Conclusions

Currently, the emerging antimicrobial resistance trends are a serious challenge to limiting the virulence properties of bacterial pathogens. Therefore, bee products are very promising natural antimicrobial agents. Bee products act against both Gram-positive and Gram-negative bacteria, as well as fungi and biofilm-forming microorganisms. Their antimicrobial activity depends on chemical composition. The most challenging problems associated with using bee products for medical purposes are dosage and safety. The complexity and variability in the composition of these products raise the need for their standardization before safe and predictable clinical uses can be achieved.

## Figures and Tables

**Table 1:** Antibacterial activity of different honeys.

Microorganism	MIC	Honey Samples	Reference
*S. aureus*	126–185 mg/mL	*Apis mallifera* honey	[37]
	12.5 mg/mL	Honey from Basrah region/Iraq	[38]
	0.625–500 mg/mL	Honey from India	[39]
	10% (*v/v*)	Honey from the Adamawa region of Cameroon.	[28]
	10–20% (*v/v*)	Manuka	[36]
	142.87–214.33 mg/mL	*Tetragonisca angustula* honey	[37]
	190 ± 10 mg/mL	Melipona honey	[30]
	≤6.25% (*v/v*)	Multifloral	[23]
*E. coli*	2.500 mg/mL	Honey from India	[19]
	6.25 mg/mL	Honey from Basrah region/Iraq	[18]
	100 mg/mL	Egyptian clover honey	[20]
	150 ± 10 mg/mL	Melipona honey	[30]
	25% (*v/v*)	Multifloral	[23]
*P. aeruginosa*	1.250 mg/mL	Honey from India	[19]
	1.5 mg/mL	Honey from Basrah region/Iraq	[18]
	10–20% (*v/v*)	Manuka honey	[11]
	≤6.25% (*v/v*)	Multifloral	[23]
*C. albicans*	40% (*v/v*)	Agastache	[15]
	40% (*v/v*)	Manuka	[15]
	25–47% (*v/v*)	Honeys from southern Iran	[14]

**Table 2:** Activity of propolis against microorganisms.

Microorganism	MIC Value mg/mL (Min.–Max.)	Geographical Origin	Reference
*S. aureus*	0.08–1.2	Europe	[48]
	0.12	Greece	[51]
	0.59–1.72	Portugal	[42]
	0.25	Poland	[46]
	0.55	Brazil	[52]
	0.382	Brazil	[53]
	0.062–>1.0	Brazil	[47]
*S. epidermidis*	0.05	Greece	[51]
	0.77	Brazil	[52]
	0.6	Europe	[48]
	0.1	Poland	[46]
*S. pyogenes*	0.51	Brazil	[54]
	0.08–0.6	Europe	[48]
	0.05	Poland	[46]
*E. faecalis*	0.88	Brazil	[52]
	1.352	Brazil	[53]
	0.512	Brazil	[54]
	0.5	Poland	[46]
	0.031–>1.0	Brazil	[47]
*E. coli*	0.40	Greece	[51]
	0.512	Brazil	[54]
	0.6–5.0	Europe	[48]
	3.19–4.94	Portugal	[42]
	5.0	Poland	[46]
*P. aeruginosa*	0.24	Greece	[51]
	5.83	Brazil	[52]
	1.56–2.81	Portugal	[42]
	0.25	Brazil	[54]
	0.6–2.5	Europe	[48]
	5.0	Poland	[46]
*E. cloace*	0.30	Greece	[51]
	>5.0	Europe	[48]
*K. pneumoniae*	3.33	Brazil	[52]
	0.512	Brazil	[54]
	5.0	Poland	[46]
	0.6–>5	Europe	[48]
*P. mirabilis*	2.25	Brazil	[52]
	0.512	Brazil	[54]
*C. albicans*	13.19–13.90	Portugal	[42]
	7.90–9.25	Brazil	[52]
	6.25	Poland	[49]
	0.3–5.0	Europe	[48]
	0.256	Brazil	[54]
	>1.0	Brazil	[47]

**Table 3:** The activity of bee venom, melittin and royal jelly against microorganisms.

Microorganism	Bee Venom	Melittin	Royal Jelly	Royalisin	Reference
*S. aureus*	10–60 µg/mL	6–10 µg/mL			[72]
	6.25 µg/mL				[73]
		2 µM			[65]
		0.5–4 µg/mL			[74]
		2–4 µg/mL			[75]
	0.7 µg/mL	3.6–57.3 µg/mL			[76]
			20–80 *w/w*		[77]
			3.4–9.0 mg/mL		[78]
			15.63–500 µg/mL		[79]
			12.5 mg/mL		[80]
				7.5 µg/mL	[81]
				<250 µg/mL	[82]
MRSA	60 µg/mL	10–100 µg/mL			[72]
	0.78–3.12 µg/mL				[73]
		1–4 µM			[65]
	0.085–0.11 µg/mL				[67]
		0.5–4 µg/mL			[74]
	7.2 µg/mL	6.7 µg/mL			[76]
			30–70 *w/w*		[77]
			8.0–14.5 mg/mL		[78]
*S. epidermidis*	60 µg/mL	10 µg/mL			[72]
	0.78 µg/mL				[73]
			40–80 *w/w*		[77]
			8.7–10.3 mg/mL		[78]
*S. saprophiticus*	10 µg/mL	10 µg/mL			[72]
*S. pyogenes*	100 µg/mL	10 µg/mL			[72]
*S. pneumoniae*	3.12 µg/mL				[73]
*S. bovis*	1.56 µg/mL				[73]
*S. oralis*	100 µg/mL	200 µg/mL			[72]
*S. salivarius*		10 µg/mL			[70]
*S. sanguinis*		10 µg/mL			[70]
*S. sorbinus*		10 µg/mL			[70]
*S. mitis*		10 µg/mL			[70]
*S. mutans*		40 µg/mL			[70]
*S. agalactiae*	40 µg/mL	30 µg/mL			[72]
	6.25 µg/mL				[72]
			50–100 *w/w*		[77]
*E. faecalis*	100–200 µg/mL	30–50 µg/mL			[72]
		1–8 µg/mL			[74]
		2–4 µg/mL			[75]
		6 µg/mL			[70]
			40–100 *w/w*		[77]
			3.7–13.7 mg/mL		[78]
*E. faecium*			50–70 *w/w*		[77]
*E. casseliflavus*	10 µg/mL	8 µg/mL			[72]
VRE	200 µg/mL	50 µg/mL			[72]
*L. monocytogenes*		2–4 µg/mL			[77]
		0.315 µg/mL			[83]
*E. coli*	60–200 µg/mL	30 µg/mL			[72]
		1–2 µM			[65]
		16 µg/mL			[75]
			60–100 *w/w*		[77]
			7.0–7.1 mg/mL		[78]
			500 µg/mL		[79]
			13.5 mg/mL		[80]
				NI	[81]
				>2000 µg/mL	[82]
*K. pneumoniae*	30–500 µg/mL				[72]
		2 µM			[65]
			80–100 *w/w*		[77]
			8.0–8.1 mg/mL		[78]
*S. choleraesuis*	500 µg/mL				[72]
				9 µg/mL	[81]
*S. flexneri*	60 µg/mL				[72]
			14.5 mg/mL		[80]
*P. aeruginosa*	500–>500 µg/mL	100 µg/mL			[72]
		2 µM			[65]
		≥64 µg/mL			[75]
		0.125–4 µg/mL			[84]
			60–100 *w/w*		[77]
			3.3–14.4 mg/mL		[78]
			15.5 mg/mL		[80]
				10 µg/mL	[81]
*A. baumannii*	30 µg/mL	30 µg/mL			[72]
		17–20 µg/mL			[84]
*A. baumannii* (XDR)		31–45.4 µg/mL			[85]
*A. baumannii* (PDR)		>284 µg/mL			[85]
*C. albicans*	60 µg/mL	100 µg/mL			[48]
	40 µg/mL				[86]
	62.5–125 µg/mL				[87]
			62.5–125 µg/mL		[79]
*C. glabrata*	>500 µg/mL	300 µg/mL			[48]
*C. parapsilosis*	60 µg/mL	100 µg/mL			[48]
*C. tropicalis*	300 µg/mL				[48]
*C. krusei*	60 µg/mL	30 µg/mL			[48]

*w/w*—weight/weight of RJ on water.

**Table 4:** Chemical structure and amino acid sequence of bioactive compounds from bee products as antimicrobial agents.

Type of Bee Product	Group/Bioactive Compound	Chemical Structure/Amino Acid Sequence
Honey	Flavonoid: luteolin	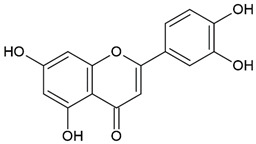
	Flavonoid: pinobanksin	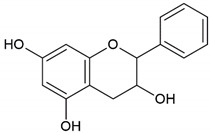
Propolis	Phenolic compound: 2,2-dimethyl-8-prenylchromene	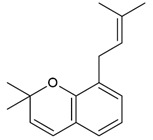
	Phenolic compound: 4-hydroxy-3,5-diprenyl cinnamic acid (artepillin C)	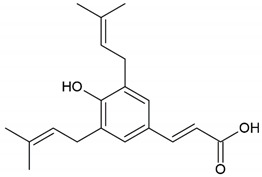
	Phenolic compound: 3-prenyl cinnamic acid allyl ester	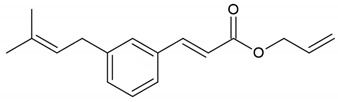
	Terpenoid: isocupressic acid, a labdane diterpenoid	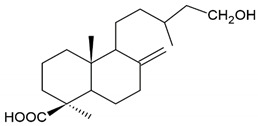
Honey, propolis	Flavonoid: apigenin	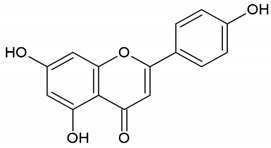
	Flavonoid: pinocembrin	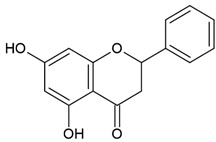
	Flavonoid: quercetin	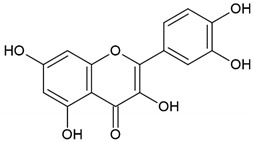
	Flavonoid: chrysin	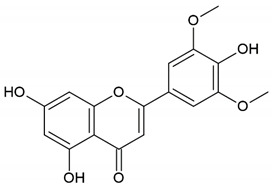
	Flavonoid: fisetin	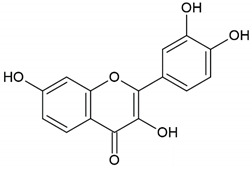
	Flavonoid: caffeic acid phenethyl ester	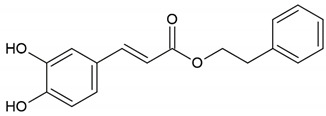
Propolis, royal jelly	10-Hydroxyl-2-decenoic acid	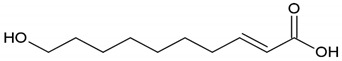
Bee venom	Melittin	GIGAVLKVLTTGLPALISWIKRKRQQ
	Apamin	CNCKAPETALCARRCQQH
	Melectin	GFLSILKKVLPKVMAHMK-N

**Table 5:** The most commonly used methods for the analysis of selected antibacterial components contained in bee products.

Bee Products	Studied Compounds	Applied Methods	Reference
Honey	Phenolic and flavonoid fraction, proline	Spectrophotometry	[120]
Honey	Phenolic fraction	Reversed-phase high-performance liquid chromatography–electrospray ionization time-of-flight mass spectrometry (RP-HPLC-ESI-TOF MS)	[124]
Honey	Phenolic fraction and sugars	Gas chromatography–mass spectrometry (GC-MS), Fourier transform infrared (FTIR) spectroscopy, X-ray diffraction	[126]
Honey	Phenolic fraction	Ultrahigh-pressure liquid chromatography–tandem quadrupole mass spectrometry (UHPLC-Q MS/MS)	[127]
Honey	Methylglyoxal	Liquid chromatography–electrospray ionization time-of-flight mass spectrometry (LC-ESI-TOF MS)	[128]
Honey	Methylglyoxal	Infrared (IR) spectroscopy	[127]
Propolis, honey, bee pollen	Phenolic fraction	Reversed-phase high-performance liquid chromatography with UV detection (RP-HPLC-UV)	[123]
Propolis	Phenolic fraction	Fourier transform infrared attenuated total reflection spectroscopy (FTIR–ATR)	[119]
Propolis	Phenolic fraction	Ultrahigh-pressure liquid chromatography with a linear ion trap–high-resolution Orbitrap mass spectrometry (UHPLC–LTQ/Orbitrap MS/MS)	[127]
Propolis	Phenolic fraction	Direct analysis in real time–Orbitrap mass spectrometry (DART-Orbitrap MS)	[128]
Propolis	Phenolic fraction	High-performance liquid chromatography–electrospray ionization mass spectrometry (HPLC-ESI-MS/MS)	[129]
Propolis	Phenolic fraction	High-performance liquid chromatography with UV detection (HPLC-UV)	[125]
Propolis	Phenolic and flavonoid fraction	Spectrophotometry and colorimetry	[121]
Royal jelly	Polyphenols	Turbulent flow chromatography–Orbitrap mass spectrometry (TFC-Orbitrap MS)	[130]
Royal jelly	10-Hydroxy-2-decenoic acid (10-HDA)	Attenuated total reflectance Fourier transform mid-infrared (ATR-FTMIR) and near-infrared (NIR) spectroscopy	[134]
Royal jelly	Phenolic and flavonoid fraction, 10-HDA	Spectrophotometry	[122]

## Data Availability

Not available.

## Sample Availability

Not available.
